# Screening, Prognostic, and Predictive Molecular Tools for Colorectal Cancer: Recent Advances in the Classical Background

**DOI:** 10.3390/ijms27052251

**Published:** 2026-02-27

**Authors:** Mihaela Cristina Pavalean, Ioana Maria Lambrescu, Mihai Ioan Pavalean, Gisela Gaina, Laura Cristina Ceafalan, Mihail Eugen Hinescu

**Affiliations:** 1 Faculty of Medicine, Carol Davila University of Medicine and Pharmacy, 050474 Bucharest, Romaniamihai-ioan.pavalean@drd.umfcd.ro (M.I.P.);; 2 Victor Babes National Institute of Pathology, 050096 Bucharest, Romania

**Keywords:** colorectal cancer, molecular tool, biomarker, screening, prognostic biomarker, predictive biomarker, emerging biomarker

## Abstract

Colorectal cancer (CRC) continues to represent a substantial worldwide health burden. Accurate risk classification and early detection have a significant impact on prognosis. There is still a significant percentage of patients who are diagnosed at advanced stages, notwithstanding the progress that has been made in screening and treatment. Thus, improved molecular tools that encompass the biological complexity of CRC are needed. High-throughput technologies have expanded the biomarker array for CRC screening, prognosis, and therapeutic prediction. This review summarizes evidence on established and emerging molecular tools from tumor tissue, blood, and stool samples, such as DNA mutations, methylation markers, RNA signatures, circulating tumor DNA (ctDNA), circulating cell-free DNA (cfDNA), extracellular vesicles, and multi-omic composite assays. These provide alternatives to conventional approaches that are relatively less invasive and more sensitive. Prognostic biomarkers—such as RAS, BRAF, HER2 alterations, mismatch repair deficiency, tumor mutational burden, methylation signatures, and non-coding RNAs—provide insight into tumor behavior and recurrence risk. To guide targeted therapies, immunotherapies, and chemotherapy response, predictive biomarkers such as RAS/BRAF mutations, HER2 amplification, MSI-H/dMMR status, POLE/POLD1 mutations, DNA methylation panels, miRNAs, lncRNAs, and liquid biopsy markers are crucial. Emerging technologies such as multi-omics, AI-enhanced biomarker discovery, and novel liquid biopsy components (evDNA, circRNAs) pave the way to precision oncology. These molecular tools have the potential to change how CRC is managed by earlier detection and more precise predictive biomarkers. However, large-scale validation and clinical standardization are still crucial for their extensive utilization.

## 1. Introduction

Colorectal cancer (CRC) is the world’s third most diagnosed cancer, being the second most common cause of cancer-related death in the world according to the global cancer statistics [1]. One representative epidemiological study on population health, the Global Burden of Disease (GBD) 2021, investigated CRC incidence, fatality, and disability-adjusted life years (DALYs) from 1990 to 2021, reporting 2,194,143 globally incident CRC cases, 1,044,072 deaths, and 24,401,100 DALYs. Data analysis highlights that from 1990 to 2021, there was a rise in the global incidence, mortality and DALYs associated with CRC, with male predominance. Moreover, there has been a progressive decline in age-standardized DALY rate (ASDR), reflecting the significant progress in CRC treatment [2]. The most recent estimates from the Global Cancer Observatory (GLOBOCAN) indicate that colorectal cancer accounted for nearly 1 million deaths and approximately 2 million new cases worldwide in 2022 [3]. Also, the incidence of early-onset CRC (individuals under 50 years old) has been increasing, especially in developed Western countries, frequently with more invasive characteristics like poorly differentiated adenocarcinoma. With an accelerated incidence rate and high mortality rate, it has evolved in the past decades into a significant global health challenge [4].

Despite some advances in early diagnosis and therapy, the prognosis for advanced CRC remains poor, with very high rates of recurrence and metastasis, and an overall five-year survival rate around 50%. This is mainly due to the asymptomatic nature of CRC in its early stages, leading to most patients being diagnosed at an advanced stage, often with metastases [5]. The underlying mechanisms of CRC initiation, progression, and invasion remain poorly understood. As a result, identifying reliable methods and biomarkers for early detection, along with gaining a deeper understanding of the molecular pathways involved in CRC development and progression, has become a central focus of current research [1,6].

CRC development is a multistep process with progressive accumulation of genetic mutations and epigenetic alterations that drive the transformation of a benign polyp into an invasive carcinoma capable of local invasion and distant metastasis. CRC arises from the interplay between genetic predisposition and environmental influences, progressing through distinct stages that culminate in the adenoma-to-carcinoma sequence [7]. Three principal molecular pathways are implicated in CRC carcinogenesis: the chromosomal instability (CIN) pathway, the microsatellite instability (MSI) pathway, and the CpG island methylator phenotype (CIMP) pathway, also referred to as the serrated pathway [6]. Also, a key determinant of cancer behavior is the tumor immune microenvironment (TME). Recent research has investigated the mechanisms by which the TME promotes colorectal cancer progression, facilitates metastasis, mediates immune tolerance, and contributes to treatment resistance. The TME represents a dynamic ecosystem composed of both cellular and extracellular components that engage in reciprocal communication, thereby influencing malignant cell proliferation, stemness, and dissemination. Key cellular constituents, including tumor-associated macrophages (TAMs), cancer-associated fibroblasts (CAFs), endothelial cells, pericytes, adipocytes, regulatory T cells (Tregs), and myeloid-derived suppressor cells (MDSCs), play essential roles in the development and maintenance of tumor vasculature and the metastatic process. Exosomes, which are vesicles released by malignant cells, fibroblasts, immune cells, and endothelial cells within the TME, also play critical roles in metastasis, immune evasion, and resistance to therapy [8].

Advances in understanding the molecular mechanisms underlying CRC have led to the development of new and less invasive screening and diagnostic tests designed to detect early neoplastic changes and even precancerous lesions. Additionally, novel prognostic and predictive molecular markers have been identified and validated, derived from specific pathways that play a critical role in tumor growth and progression [9]. Various biomarkers are already available, starting from tumor biopsy but also from other biological samples, with blood, stool, saliva, and urine emerging as promising and less invasive sources. Stool and blood have been the primary biospecimens studied for early CRC detection, while tumor tissue and blood are most routinely used for prognosis and predicting clinical outcomes. Due to their ability to reveal tumor heterogeneity, liquid biopsies have emerged as powerful biomarker sources not only for screening and diagnostic purposes, but also as valuable tools for monitoring disease progression and therapeutic response [10]. Such biomarkers are vital for guiding treatment decisions in patients with locally advanced or metastatic colorectal cancer (mCRC) and those who develop treatment resistance [6].

The integration of high-throughput molecular technologies with multi-omic data, clinical information, and epidemiologic context—leveraged through artificial intelligence—will enhance biomarker discovery and support more precise clinical decision-making [11].

This review aims to summarize both established and emerging biomarkers with good clinical performance as early diagnosis and prognostic tools, focusing on less invasive and more specific tests, with an acceptable cost.

## 2. Molecular Tools for Screening

Colorectal cancer (CRC) is one of the leading causes of cancer-related morbidity and mortality worldwide, and its prognosis is strongly dependent on early detection [12]. Traditional screening methods such as colonoscopy and fecal occult blood testing, while effective, have limitations related to invasiveness, compliance, and sensitivity [13]. In recent years, advances in molecular biology have provided novel non-invasive or minimally invasive tools that complement standard approaches and improve early detection [14].

Molecular screening tools for CRC are based on identifying genetic, epigenetic, and transcriptomic alterations characteristic of tumor development. These include DNA mutation assays (e.g., KRAS, APC, TP53 mutations) [12], DNA methylation markers (such as SEPT9, used in blood-based assays) [13,15], RNA and microRNA expression profiles [16] and circulating tumor DNA (ctDNA) detection [6,12]. In addition, multitarget stool DNA tests combine mutation and methylation analysis with hemoglobin immunoassays to enhance sensitivity [17]. The integration of next-generation sequencing (NGS) and digital PCR technologies has further increased the accuracy of detecting rare tumor-derived nucleic acids in blood or stool samples [16].

Molecular tools are a significant step toward precision screening tests for colorectal cancer (CRC) due to their high specificity and potential for non-invasive use. These strategies aim to improve patient adherence, identify precancerous lesions earlier, and eventually lower mortality [6].

### 2.1. Stool-Based Tests

Stool tests for colorectal cancer (CRC) screening seek to identify biomarkers—such as bleeding, mutant DNA, methylation DNA, and tumor-associated proteins—that are released into the stool by neoplastic lesions prior to the onset of clinical signs [18,19]. While more recent molecular tests identify DNA changes or other tumor indicators, traditional stool tests identify occult blood. Numerous stool-based screening techniques, each with unique mechanisms, advantages, and disadvantages, are currently in use or being developed (Table 1).

Stool-based tests remain non-invasive, convenient, and generally more acceptable to patients compared to colonoscopy [31]. The classic gFOBT uses a guaiac paper reagent that turns blue in the presence of heme peroxidase activity, but its sensitivity is modest and dietary interference is a challenge [20]. The FIT specifically targets human hemoglobin, improving sensitivity and specificity over gFOBT and reducing dietary confounding [20]. The M2-PK stool test detects a glycolytic enzyme isoform (M2-pyruvate kinase), aiming to capture metabolic changes in tumor cells, and is independent of bleeding. Reported performance varies, but meta-analyses suggest potential utility as a complementary test [23].

The mt-sDNA test (for example, Cologuard^®^) combines assays for methylated genes (e.g., NDRG4, BMP3), mutant KRAS, a reference human DNA marker (e.g., β-actin), and fecal hemoglobin, producing a composite risk score (https://www.cms.gov/medicare-coverage-database CAG-00440N, accessed on 20 December 2025). In a large comparative trial, the mt-sDNA test showed higher sensitivity than FIT for detecting colorectal cancer and advanced adenomas, albeit with somewhat lower specificity [24]. Moreover, a next-generation multitarget stool DNA test, Cologuard plus^®^, also approved by FDA for screening in 2024, showed better results in a large observational study. It significantly outperformed an independent FIT test for overall CRC sensitivity and sensitivity to advanced precancerous lesions, but the specificity was lower [25].

More recently, stool DNA methylation panels focusing on one or a few CpG loci (e.g., methylated SDC2, SHOX2) have been explored to reduce cost and complexity while retaining diagnostic performance [32].

Several studies have demonstrated the diagnostic potential of fecal miRNA profiling. Early reports identified elevated stool levels of miR-92a and miR-21 in CRC patients compared with controls, achieving sensitivities ranging from 71 to 89% and specificities between 65 and 81% [33]. More recently, multicenter next-generation sequencing approaches confirmed that stool miRNA signatures can robustly discriminate CRC from adenomas and healthy individuals, providing reproducible molecular markers across populations [28]. Beyond miRNAs, host mRNA transcripts have been explored as biomarkers. A phase 3 trial validated a multitarget stool RNA (mt-sRNA) panel, which integrates multiple gene expression markers with clinical variables, and demonstrated noninferior performance compared to colonoscopy for advanced neoplasia detection, with sensitivities exceeding 80% for CRC and approximately 50% for advanced adenomas [34]. Analytical validation further confirmed the reproducibility and stability of RNA detection in stool samples [30].

In summary, stool-based CRC screening tests can be broadly categorized by target (blood, DNA mutation, DNA methylation, metabolic enzyme), and each type represents a trade-off between sensitivity, specificity, cost, and operational complexity. Combining or layering these assays may improve detection of early and non-bleeding lesions, but practical implementation must balance performance with affordability and adherence.

### 2.2. Blood-Based Tests

The objective of blood-based tests for colorectal cancer screening is to provide a less intrusive substitute for endoscopic or stool-based methods. They identify tumor-derived genetic, epigenetic, or proteomic changes, as well as circulating tumor cells (CTCs) and cell-free DNA (cfDNA) released into the bloodstream [6] (Table 2).

#### 2.2.1. Epigenetic Biomarkers in cfDNA-Based Colorectal Cancer Screening

Particularly promising among blood-based screening methods are epigenetic assays that target DNA methylation. The aim of these tests is to detect hypermethylation of tumor suppressor gene promoter regions in the bloodstream as cell-free DNA (cfDNA) [40]. Aberrant DNA methylation, which occurs frequently and at early stages of colorectal carcinogenesis, represents a promising biomarker for early detection [41]. Principal methylation targets include SEPT9, NDRG4, BMP3, SDC2, SHOX2, and VIM, which exhibit inconsistent diagnostic efficacy across study results [42]. The first FDA-approved blood-based assay for colorectal cancer screening is the Epi proColon^®^ test (Epigenomics AG, Berlin, Germany), which measures plasma methylated SEPT9 [43]. SEPT9, also known as Septin9, is one of numerous genes involved in controlling cytokinesis and cellular proliferation. It also regulates membrane reconstruction, vesicle trafficking, cell polarization, and cytoplasmic division [44]. The development of colorectal cancer is therefore caused by methylation of the SEPT9 gene, which sets off cellular progression towards malignancy in the colon mucosa [45].

According to reported performance metrics of the Epi proColon^®^ test, the detection of colorectal cancer in average-risk individuals has a specificity of 80–90% and a sensitivity of approximately 68–72% (https://www.cms.gov/medicare-coverage-database/view/ncacal-decision-memo.aspx?NCAId=299, accessed on 15 December 2025). This method’s main benefits are its non-invasiveness, simplicity in sample, and ability to increase compliance among those who are reluctant to undergo a colonoscopy or participate in stool-based testing. Nonetheless, current limitations include limited sensitivity for advanced adenomas, high costs, and a risk of false negatives in early stages. These issues highlight the need for improved biomarker development and better test optimization [6].

#### 2.2.2. Mutation-Based cfDNA Assays in Colorectal Cancer Screening

Another possible class of blood-based colorectal cancer screening techniques is mutation-based diagnostics, which detect tumor-specific somatic mutations in circulating tumor DNA (ctDNA) or cell-free DNA (cfDNA) released into the bloodstream. These assays typically focus on recurrent driver mutations in genes essential for colorectal tumor development, including KRAS, APC, TP53, and BRAF [46]. To facilitate highly specific detection of tumor-derived genetic alterations in plasma, advanced next-generation sequencing (NGS) panels have been developed to simultaneously interrogate multiple mutation hotspots [39]. These assays show significant potential for non-invasive screening and early detection. Currently, however, their primary use remains in research settings or in monitoring patients after diagnosis for disease burden and treatment response [39]. Detecting minimum residual disease (MRD) after curative resection and guiding treatment options by identifying actionable mutations are key benefits of mutation-based cfDNA screening [47]. Yet, the scant ctDNA traces in early-stage colorectal cancer pose a significant hurdle, dulling the sharpness of screening and making it harder to detect the disease in people without symptoms [48]. Furthermore, ctDNA is rarely used for early tumor screening since early-stage tumor cells undergo minimal necrosis and release very little ctDNA into the bloodstream [49]. Therefore, the main goal of current research is to improve analytical sensitivity through the use of ultra-deep sequencing, error-suppression techniques, and fragmentomics-based enrichment procedures.

#### 2.2.3. Protein-Based Biomarkers in Colorectal Cancer Screening

Protein-based biomarkers are one of the oldest and most extensively studied blood-derived indicators of CRC. These tests detect variations in blood protein concentrations associated with malignancies. Such alterations may come from direct tumor development or systemic consequences after malignant transformation [37]. The following are examples of biomarkers that are often evaluated: carcinoembryonic antigen (CEA), carbohydrate antigen 19-9 (CA19-9), cancer antigen 125 (CA125), tissue factor pathway inhibitor-2 (TFPI2), and angiopoietin-like protein 2 (ANGPTL2) [50,51,52]. For monitoring recurrence after curative resection, CEA is particularly well established [53]. Similar proteins’ broad clinical availability, affordability, and simplicity of testing make them useful instruments for postoperative surveillance and illness monitoring [37]. However, because protein levels can also rise in benign or inflammatory conditions, their use in primary CRC screening is limited by low sensitivity and specificity, especially in early-stage disease [54]. Current research aims to improve diagnostics by integrating serum protein markers with genetic and epigenetic signatures into multi-analyte liquid biopsy platforms. This approach seeks greater sensitivity while maintaining cost-effectiveness [55]. For example, in a pan-cancer context including CRC, the CancerSEEK test shows better cancer diagnosis performance than single-analyte techniques by combining the detection of circulating tumor DNA mutations with the measurement of certain serum proteins, such as CEA, CA-125, CA19-9, and others.

In conclusion, only CEA is accepted in standard clinical practice, primarily for prognosis, post-treatment surveillance, and response monitoring in metastatic disease, despite numerous serum protein markers, including CA19-9, CA125, TIMP-1, TFPI2, and other inflammatory or angiogenic proteins, having been thoroughly investigated in CRC. In contrast, major oncology guidelines state that protein biomarkers other than CEA should not be used for CRC screening, diagnosis, or staging. For example, ASCO found no evidence to suggest using CA19-9 for any of these purposes in colorectal cancer [56].

This is supported by recent reviews of guideline-based practice, which show that CEA remains the only widely accepted biomarker for surveillance frameworks. Other protein markers, on the other hand, have not been recommended by guidelines because of biological confounding (benign/inflammatory conditions), lack of standardized clinical cut-offs, and limited sensitivity/specificity in early disease [57,58]. Rather than serum protein indicators, blood-based techniques have been the main focus of recent FDA approvals for CRC screening (https://www.fda.gov/medical-devices/recently-approved-devices/shield-p230009?utm, accessed on 20 December 2025). Protein biomarkers other than CEA should thus be considered as alternative or exploratory options at this stage. 

#### 2.2.4. Combined Multi-Analyte (Multi-Omics) Assays in Colorectal Cancer Screening

By incorporating serum protein markers alongside genetic and epigenetic signatures into multi-analyte liquid biopsy systems, current studies aim to enhance diagnosis [59]. This strategy aims to increase sensitivity without sacrificing cost-effectiveness. These systems aim to enhance early-stage detection and overcome the sensitivity limitations of single-analyte assays by integrating complementary molecular markers [59]. The Guardant Shield™ assay (Guardant Health) exemplifies a composite risk score for colorectal cancer detection by using cfDNA methylation profiling, fragmentomic patterns, and mutation analysis [38]. Comparably, the ColoDefense test has shown higher sensitivity than single-gene methylation tests by using dual-target methylation analysis of SEPT9 and SDC2 in plasma [36]. Validation studies have demonstrated that a specificity of approximately 90% and sensitivities exceeding 85% have been attained in certain multi-analyte assays for CRC detection. This indicates that there is significant potential for population-level screening [55]. These tests are still being evaluated for large-scale implementation, despite the promising results, due to the ongoing challenges of cost, accessibility, and assay standardization [55,60]. However, non-invasive, accurate, and comprehensive colorectal cancer screening methods have taken a giant leap forward with the incorporation of multi-omics biomarkers (Table 2).

#### 2.2.5. Current Research Trends in Blood-Based Colorectal Cancer Screening

The integration of sophisticated computational and molecular approaches to enhance diagnostic accuracy is a growing focus of recent research in blood-based CRC screening. The use of fragmentomic analysis and machine learning techniques to improve the identification of weak cfDNA signals coming from extremely small or early-stage tumor lesions is one significant area of advancement [61]. By using fragment size distributions, nucleosome positioning signals, and genome-wide cfDNA fragmentation patterns—all of which are detectable even at very low tumor-derived DNA fractions—fragmentomic approaches may be able to outperform conventional mutation-based diagnostics. In cases when mutation-based ctDNA detection may not be enough, this increases sensitivity in early-stage malignancies [61,62,63].

Integrating multiple weak signals—such as methylation profile, fragmentomic signatures, mutation load, and clinical variables—into composite prediction models, machine learning techniques further improve diagnostic performance. When compared to single-marker tests, these integrated methods have shown better early-stage detection accuracy, especially in low-tumor-fraction circumstances [64].

Concurrently, multi-cancer early-detection tests are being developed to identify patterns of DNA methylation and fragmentation specific to colorectal cancer and other malignancies. In extensive cohort studies, the Galleri^®^ test (GRAIL Inc., Menlo Park, CA, USA) showed promising sensitivity for colorectal cancer. It uses cfDNA methylation patterns to identify over fifty cancer types [65]. A wave of health-economic and population-based studies is unfolding in tandem with rapid technological progress. These efforts aim to uncover how cost-effective, practical, and widely accepted blood-based assays could be if introduced into real-world screening programs, and how they compare with tried-and-true methods such as the fecal immunochemical test or colonoscopy [60]. These efforts highlight the emerging understanding that accessibility, cost, and patient adherence—in addition to analytical performance—will be necessary for the effective incorporation of liquid biopsy into population screening programs.

Nanoscale imaging technologies use the mechanical properties of colorectal cancer cells. For example, atomic force microscopy (AFM) micro- and nano-indentation methods allow precise measurement of these properties. These methods are increasingly used in cancer research. AFM-force spectroscopy (AFM-FS) is a promising strategy for detecting and monitoring cancer by analyzing the mechanical properties of circulating tumor cells. Monitoring dynamic biomarker changes at the nanoscale enables more accurate diagnoses. Data from AFM-based techniques help explain the molecular mechanisms behind disease initiation and progression. Understanding these mechanisms may support identifying biomarkers to evaluate therapies [66]. Moreover, this new technique has demonstrated additional applications in CRC. A recent study analyzing tissue rigidity in clinical samples using AFM demonstrates a relationship between tissue stiffness and genetic factors. Findings show that gene mutations can modify tissue mechanical properties and activate specific pathways, especially in KRAS-mutated tumors, which exhibit greater stiffness than non-mutated tumors. The results further reveal that colon tissue is initially soft and becomes progressively stiffer during carcinogenesis, particularly within stromal regions. However, to confirm these observations, additional studies in larger cohorts are needed. Nevertheless, studies suggest that potential drugs targeting tumor stiffness could improve treatment access in RAS-mutated CRC [67].

Importantly, in addition to analytical performance, practical implementation issues must also be addressed. Incidental findings, such as signals of ambiguous clinical significance or cancer signals without an identifiable tumor site, may be produced by multi-analyte blood-based screening assays [64]. These findings can result in patient anxiety and require additional diagnostic tests. Moreover, false-positive findings may require subsequent procedures, such as imaging, colonoscopy, or repeated molecular testing, thereby increasing the demand for healthcare services. To sum up, the effective use of highly sensitive molecular screening techniques requires well-planned diagnostic procedures and health-economic analyses that balance the advantages of early detection against the potential risks of overdiagnosis and unnecessary investigations [68].

#### 2.2.6. Blood-Based Tests Have Not Yet Been Incorporated into Clinical Guidelines for First-Line Colorectal Cancer Screening

A blood-based test could enhance screening compliance, enable earlier detection of colorectal cancer, and contribute to lowering mortality associated with the disease. Detecting ctDNA in early-stage colorectal cancer or precancerous lesions can be challenging due to the low quantity of DNA shed by tumor cells. To enhance sensitivity, several approaches are being explored, including the use of CRC-specific DNA methylation markers, fragmentomics, and proteomics.

## 3. Prognostic Molecular Markers for CRC

### 3.1. General Considerations on Molecular Prognostic Tools in CRC

The idea of molecular prognostic tools in CRC stems from the understanding that tumor behavior is not only influenced by its anatomical and histological characteristics, but also by complex molecular processes that drive malignancy. Over the last 20 years, advances in genomics, transcriptomics, epigenomics, and proteomics have enabled a deeper understanding of these mechanisms, leading to the discovery of molecular changes that may serve as prognostic or predictive biomarkers. Molecular prognostic tools in CRC arise from understanding that tumor behavior depends not only on anatomical and histological features, but also on molecular processes driving malignancy.

For a long time, clinicopathological parameters—most notably the tumor–node–metastasis (TNM) classification systems developed by the UICC and the AJCC—have served as the basis for CRC prognosis. By evaluating the anatomical extent of disease, the TNM Classification of Malignant Tumors, 9th Edition [69], remains the foundation for staging and treatment planning.

Nevertheless, the TNM system may not always adequately reflect the biological diversity and varying clinical behaviors observed within individuals classified by the same stage, even though it is widely used. As a result, molecular predictive markers have become essential supplementary instruments in this context.

These biomarkers, which indicate the tumor’s biological status, are associated with medical outcomes such as disease-free survival (DFS), overall survival (OS), and the risk of recurrence.

In contrast to predictive biomarkers, the primary function of prognostic biomarkers is to provide intrinsic information about the expected course of disease, independent of treatment. When used with existing staging frameworks, molecular prognostic markers may improve risk classification and provide a more personalized approach to patient care in colorectal cancer (CRC) [70].

A biomarker must show a high correlation with treatment-independent clinical outcomes, and its presence or absence must not impact the efficacy of therapy in order to be deemed strictly prognostic. The combined predictive and prognostic potential of specific biomarkers should not be ignored [11].

Independent of particular treatment approaches, prognostic indicators provide crucial information about the course and probable fate of colorectal cancer. Risk classification, treatment intensity decisions, follow-up strategies, and patient counseling are all greatly impacted by them. Differentiating between prognostic markers—which indicate how aggressive the disease is and how long patients can expect to live—and predictive markers—which indicate how likely a patient is to respond to a given therapy—is crucial [70].

### 3.2. Recent Advances in Molecular Prognostic Tools

Building on these classical molecular pathways, recent technological advances have led to the development of integrative prognostic models that combine genomic, transcriptomic, epigenetic, proteomic, and even metabolomic data. These multi-omic approaches aim to capture the full molecular complexity of CRC, providing a more comprehensive understanding of tumor biology and improving the accuracy of prognostic assessment. Unlike traditional single-marker analyses, which offer limited predictive power, multi-omic signatures account for interactions across multiple biological layers, yielding robust molecular classifiers with direct clinical relevance.

#### 3.2.1. Clinically Relevant Genomic Alterations in CRC

Genomic profiling has identified key somatic mutations and copy number variations that correlate with disease aggressiveness and clinical outcome.

For example, mutations in the RAS family (mainly KRAS [71] and NRAS [72,73]) and BRAF have important prognostic and therapeutic implications, particularly in metastatic CRC (mCRC) [74]. Current international guidelines recommend comprehensive RAS mutation testing for all patients with metastatic colorectal cancer (mCRC), primarily to guide the use of anti-EGFR therapy.

The prognostic value of *KRAS* and *BRAF* mutations in localized CRC is less clearly defined. However, a recent pooled analysis from seven major clinical trials (ACCENT/IDEA) evaluating stage III resectable CRC demonstrated that the presence of either *KRAS* or *BRAF* mutations was significantly associated with reduced overall survival, reinforcing the importance of incorporating molecular testing into (neo)adjuvant treatment planning [75].

In addition, *HER2* amplification or mutation, although relatively uncommon in CRC, has emerged as a relevant biomarker in a subset of tumors—particularly those that are *RAS* and *BRAF* wild-type. *HER2*-positive CRCs tend to exhibit an aggressive phenotype and may respond to HER2-targeted therapies, such as trastuzumab-based combinations [76]. These findings highlight the expanding role of genomic profiling not only for prognostication but also for optimizing therapeutic strategies in CRC. According to the 2024 ESMO consensus, testing for RAS, BRAF, and HER2 alterations is recommended for all patients with metastatic CRC to guide both prognostic evaluation and targeted therapy selection [77,78].

#### 3.2.2. DNA Repair and Instability Biomarkers

Following the identification of key oncogenic mutations such as *RAS*, *BRAF*, and *HER2*, considerable attention has turned toward biomarkers reflecting genomic stability and DNA repair capacity. These molecular features not only influence tumor aggressiveness and metastatic potential but also determine sensitivity to specific therapeutic agents, particularly immunotherapies. Alterations in DNA repair mechanisms give rise to distinct patterns of genomic instability that carry prognostic and predictive implications in CRC.

One of the most clinically significant biomarkers in this category is mismatch repair deficiency (dMMR), which leads to MSI—a phenotype resulting from the loss of function in one or more DNA mismatch repair (MMR) genes (*MLH1*, *MSH2*, *MSH6*, *PMS2*) [79,80]. Approximately 15% of all CRCs exhibit MSI, with the majority of sporadic cases arising from epigenetic silencing of *MLH1* due to promoter hypermethylation. MSI-high (MSI-H) tumors are characterized by an accumulation of insertion–deletion mutations in repetitive DNA sequences, resulting in a high neoantigen burden and increased tumor immunogenicity [81].

From a prognostic perspective, MSI-H and dMMR status are generally associated with a favorable outcome in localized disease, particularly in stage II CRC, where such tumors have lower recurrence rates and improved overall survival compared to microsatellite-stable (MSS) tumors [82]. This improved prognosis is thought to result from enhanced immune surveillance due to the elevated neoantigen load. However, in advanced or metastatic CRC, the prognostic advantage may diminish or even reverse, especially in tumors co-harboring *BRAF* mutations or exhibiting a CpG island methylator phenotype (CIMP) [82].

Consequently, MSI status now serves as both a prognostic biomarker (in early-stage disease) and a predictive biomarker (for immunotherapy responsiveness). MSI testing is now mandatory in all newly diagnosed CRCs according to NCCN and ESMO guidelines, reflecting its dual prognostic and predictive relevance [83,84].

Another important biomarker related to genomic instability is the tumor mutational burden (TMB), which quantifies the total number of somatic mutations per megabase of the coding genome. Although closely related to MSI, TMB provides a broader measure of overall genomic alteration and elevated TMB levels have been correlated with improved outcomes in subsets of CRC, particularly those exhibiting MSI-H or POLE/POLD1 mutations, which cause an ultra-mutated phenotype [85,86]. Nevertheless, the prognostic utility of TMB in microsatellite-stable tumors remains under investigation.

Together, dMMR, MSI, and TMB exemplify how the degree of genomic instability can serve as a window into both tumor biology and therapeutic opportunity. As testing technologies continue to evolve, these biomarkers are likely to play an increasingly central role in refining prognostic assessment and guiding treatment strategies for CRC patients [87].

#### 3.2.3. Epigenetic and Transcriptomic Biomarkers

Another important layer of prognostic information in CRC is provided by epigenetic and transcriptomic changes, in addition to genomic mutations and DNA repair deficiencies. Tumor behavior, metastatic potential, and treatment response are all impacted by these systems, which control gene expression without changing the underlying DNA sequence. Through aberrant DNA methylation, histone modification, and non-coding RNA production—each of which alters gene expression crucial for cell growth and differentiation—epigenetic dysregulation plays a key role in the development and progression of colorectal cancer (CRC) and offers promising targets for prognostic and therapeutic investigation [88,89].

Among epigenetic changes, DNA methylation is the most extensively studied. Global hypomethylation contributes to chromosomal instability, while promoter hypermethylation leads to silencing of tumor suppressor genes such as *MLH1* [90], *CDKN2A (p16)* [90], and *IGFBP3* [91]. The CpG island methylator phenotype (CIMP) represents a distinct subgroup of CRC characterized by widespread promoter methylation, which frequently overlaps with *BRAF*-mutated and MSI-high tumors [90]. CIMP-high cancers are often located in the proximal colon and display unique clinical and pathological features. The prognostic value of CIMP remains under debate; however, there is increasing evidence that specific methylation signatures may predict recurrence and overall survival, especially when combined with other molecular factors.

In colorectal cancer (CRC), non-coding RNAs (ncRNAs) such as microRNAs (miRNAs) and long non-coding RNAs (lncRNAs) have become important regulators of gene expression networks at the transcriptome level [92,93]. Among these, microRNAs (miRNAs) are small,~22-nucleotide RNA molecules that modulate post-transcriptional gene expression by targeting messenger RNAs (mRNAs) for degradation or translational repression [94]. Aberrant expression of specific miRNAs has been linked to tumor progression and prognosis. For instance, miR-21 [95], miR-92a [96], and miR-200c [97] are consistently upregulated in CRC and associated with poor outcomes, advanced stage, and resistance to chemotherapy. Conversely, reduced levels of miR-143 and miR-145 are associated with tumor aggressiveness and decreased survival, supporting their tumor-suppressive role [98]. Long non-coding RNAs (lncRNAs) are transcripts longer than 200 nucleotides that regulate chromatin architecture, transcription, and post-transcriptional processes.

Dysregulated lncRNAs, such as *HOTAIR*, *MALAT1*, and *CCAT1*, have been shown to promote epithelial–mesenchymal transition, as well as invasion and metastasis in CRC [99,100]. Furthermore, elevated expression of *HOTAIR* and *MALAT1* correlates with poor prognosis, advanced stage, and shorter overall survival, suggesting that these molecules are potential prognostic markers and therapeutic targets [101].

Integrating epigenetic and transcriptomic signatures into prognostic models could enhance accuracy beyond single-gene or mutation-based analyses. These biomarkers reflect the active regulatory landscape of tumor cells, providing insights into tumor plasticity and adaptability, key determinants of recurrence and treatment resistance [102]. Emerging technologies, including RNA sequencing, methylation arrays, and single-cell transcriptomics, are expected to further refine these strategies and pave the way for their inclusion into clinical prognostic algorithms for CRC [103,104].

#### 3.2.4. Liquid Biopsy Biomarkers

In recent years, liquid biopsy has emerged as a transformative approach in oncology, offering a minimally invasive method for detecting and monitoring molecular alterations in real time. Unlike traditional tissue biopsies, which provide a static snapshot of a single tumor region, liquid biopsies can capture the dynamic and heterogeneous molecular profile of CRC by analyzing tumor-derived material circulating in peripheral blood. This approach provides valuable prognostic information, facilitates early detection of recurrence, and enables longitudinal assessment of treatment response.

The principal components analyzed in liquid biopsy include cell-free DNA (cfDNA), circulating tumor DNA (ctDNA), circulating tumor cells (CTCs), and extracellular vesicles such as exosomes. Among these, ctDNA—representing tumor-derived fragments of cfDNA released into the bloodstream through apoptosis, necrosis, or active secretion—has shown the greatest promise for clinical application in CRC. Quantitative and qualitative analysis of ctDNA provides insight into tumor burden, mutational landscape, and clonal evolution over the disease course [102,105].

From a prognostic perspective, ctDNA is an indicator of minimal residual disease (MRD) and recurrence risk after curative-intent surgery or adjuvant chemotherapy. MRD detection through postoperative ctDNA analysis has emerged as one of the most powerful independent prognostic factors in CRC, outperforming traditional clinicopathologic parameters in predicting relapse [106].

Studies have consistently demonstrated that patients with detectable postoperative ctDNA have a markedly higher likelihood of disease relapse compared to those with undetectable ctDNA, often months before radiologic evidence of recurrence [107,108,109]. This makes ctDNA one of the most powerful emerging biomarkers for dynamic risk assessment and surveillance in CRC [107]. Postoperative ctDNA positivity typically precedes radiologic recurrence by 8–12 months, underscoring its prognostic utility for early relapse detection [110,111]. 

Technological advances such as digital droplet PCR (ddPCR) [112] and next-generation sequencing (NGS) [113] have enhanced the sensitivity and specificity of ctDNA detection. Analytical sensitivity varies depending on the detection platform (ddPCR versus NGS-based assays), sequencing depth, and error-correction strategies; therefore, limit of detection (LOD) values are not directly comparable across studies. Published data indicate that LOD ranges from approximately 0.01–0.1% in variant allele frequency (VAF) for ddPCR assays, 0.5–1% for standard NGS platforms, and can reach ~0.1% or lower with ultra-deep sequencing combined with advanced error-suppression techniques. Comprehensive genomic profiling assays, including commercial platforms like *Guardant360©*, can simultaneously evaluate multiple genes and genomic alterations—such as KRAS, BRAF, HER2, and PIK3CA—from a simple blood sample [114]. These analyses not only provide prognostic information but also identify actionable mutations for targeted therapy selection [115].

In addition to ctDNA, total cell-free DNA (cfDNA) levels may also have prognostic relevance, as elevated cfDNA concentrations correlate with tumor stage, metastasis, and poor survival outcomes [116,117]. However, cfDNA is a less specific marker since it includes DNA fragments from both tumor and non-tumor sources. Because cfDNA long fragments are mostly derived from cancer cells, whereas those from non-malignant cells are short, recent studies have shown that the cfDNA integrity index, defined as the ratio of long to short DNA fragments, could be a promising biomarker for early CRC diagnosis [118]. Moreover, the cfDNA and cfDNA integrity index have also shown great potential for prognosis, by detecting minimal residual disease and assessing recurrence risk [119].

The integration of liquid biopsy biomarkers into clinical practice holds considerable promise for the personalized management of CRC. Their ability to detect residual disease, predict recurrence, and monitor treatment response in a non-invasive and repeatable manner makes them invaluable tools for future precision oncology [120]. Ongoing prospective trials (UMIN000039205 (https://center6.umin.ac.jp/cgi-open-bin/icdr_e/ctr_view.cgi?recptno=R000044197, accessed on 20 December 2025); NCT05174169) are expected to establish standardized thresholds and protocols for ctDNA-guided therapy, potentially transforming postoperative surveillance and adjuvant treatment decision-making in CRC. An interim analysis from the CIRCULATE-Japan GALAXY prospective study (UMIN000039205 (https://center6.umin.ac.jp/cgi-open-bin/icdr_e/ctr_view.cgi?recptno=R000044197, accessed on 23 February 2026)), which included stage II —IV CRC patients, reported a significant improvement in overall survival (OS) and disease-free survival (DFS) in patients with ctDNA clearance after adjuvant therapy. These findings support the utility of ctDNA evaluation at diagnosis and longitudinal monitoring in CRC management [121]. Moreover, interim results from the BESPOKE prospective study (NCT04264702 (https://clinicaltrials.gov/study/NCT04264702, accessed on 23 February 2026)) reported that adjuvant therapy based on post-surgical ctDNA detection in stage II/III CRC patients improved disease-free survival (DFS) [122,123].

### 3.3. Clinical Integration, Limitations, and Future Perspectives

In the future, AI and machine learning will enable the integration of multi-omic datasets, digital pathology, and radiomic features into a single predictive model. This will change the prognostic picture of CRC.

**Figure 1:**
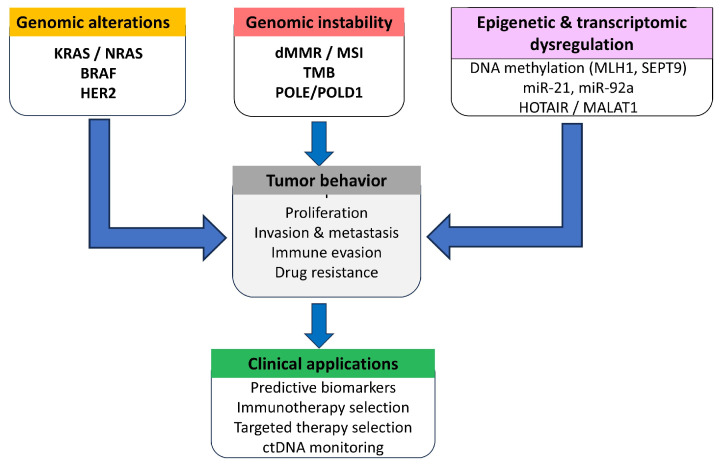
This schematic illustrates the complex mechanisms behind tumor development and behavior, which may offer valuable prognostic information and help predict treatment outcomes in colorectal cancer.

Continuous monitoring of minimum residual disease and recurrence risk is made possible by liquid biopsy techniques, especially circulating tumor DNA (ctDNA), which connects genetic findings to dynamic patient management. Advances in multi-omic profiling, single-cell sequencing, and spatial transcriptomics are expected to produce more advanced prognostic classifiers that account for both tumor biology and microenvironmental context. Ultimately, rather than only improving risk stratifications, the ability of molecular prognostic approaches to enhance patient outcomes will determine their practical relevance. Oncologists, molecular pathologists, and computational biologists will need to work together in an interdisciplinary effort to provide technology accessibility, rigorous validation, and the next step in advancement. By integrating molecular complexity into everyday clinical practice, CRC care can move decisively toward truly personalized, evidence-based prognostication (Figure 1).

## 4. Predictive Molecular Markers for CRC

### 4.1. The Utility of Predictive Tools in CRC

Recent advancements in molecular oncology have led to the identification of predictive biomarkers that help select appropriate treatments, assess prognosis, and anticipate therapy responses. These molecular tools enable clinicians to tailor treatment strategies to individual patients, enhancing therapeutic effectiveness while minimizing unnecessary interventions.

Predictive molecular tools have changed the treatment of colorectal cancer (CRC), enabling personalized therapies tailored to the tumor’s biological profile. Molecular key tests—such as RAS, BRAF, MSI, TMB, and ctDNA—are now guiding clinical decisions and treatment selection (Figure 1).

The American Society of Clinical Oncology (ASCO) recommendation for first- and second-line treatments for metastatic colorectal cancer (mCRC) includes immunotherapy for tumors with microsatellite-instability-high (MSI-H) status, and chemotherapy combined with either anti-vascular endothelial growth factor (VEGF) or anti-epidermal growth factor receptor (EGFR) therapy for microsatellite-stable (MSS) tumors [124]. In 2020, the U.S. Food and Drug Administration (FDA) approved the immune checkpoint inhibitor pembrolizumab as a first-line treatment for patients with metastatic MSI-H colorectal cancer. Furthermore, neoadjuvant immunotherapy has demonstrated promising efficacy in the management of early-stage colorectal cancer [125].

Third-line or later (salvage) therapies, such as regorafenib and trifluridine/tipiracil, offer limited clinical benefit following fluoropyrimidine-based therapy [124]. For patients with BRAF V600E-mutant tumors, combination BRAF and EGFR inhibition is the standard second-line treatment approach. Additionally, human epidermal growth factor receptor 2 (HER2) overexpression or amplification in mCRC has been linked to resistance to anti-EGFR therapy and is associated with a poorer prognosis compared to HER2-negative cases [126].

Consequently, extensive research has focused on identifying predictive biomarkers to enable pretreatment risk stratification, distinguishing patients most likely to benefit from therapy while minimizing potential harm (responders). Importantly, recognizing non-responding patients allows for avoiding unnecessary exposure to ineffective treatments [127]. Therefore, over the past years, significant research efforts have focused on employing genomic approaches to identify genomic and epigenomic signatures for prognostic evaluation. Given that fewer than 5% of transcriptionally activated genes are ultimately translated into proteins, epigenomic signatures may offer greater predictive value than purely genomic analyses [127].

### 4.2. Recent Advances in Identifying Predictive Molecular Markers with Clinical Appliance

#### 4.2.1. Genomic Biomarkers

Precision medicine plays a key role in guiding patient selection for targeted therapies and immunotherapies in metastatic colorectal cancer (mCRC) based on the presence or absence of specific genomic biomarkers.

##### KRAS Mutations as Predictive Biomarkers

Among the various genetic alterations linked to colorectal cancer (CRC), mutations in the KRAS gene have particular clinical significance, as they are associated with unfavorable prognosis and are potential therapeutic targets [128]. Mutations in the RAS gene family, including KRAS and NRAS, are seen in about 50% of CRC patients and are predictors for anti-epithelial growth factor receptor treatment (anti-EGFR) [129].

Identifying KRAS hotspot mutations is an essential component of the diagnostic evaluation, as RAS/RAF mutations confer clinical resistance to first-line EGFR-targeted antibodies and are associated with lower response rates to third-line treatment with trifluridine/tipiracil (FTD/TPI). Notably, KRAS mutations are detected in approximately 30–45% of mCRC patients [130].

Therefore, testing for RAS gene mutation status prior to chemotherapy for advanced and recurrent CRC is recommended and widely performed to assess the utility of anti-EGFR monoclonal antibodies (cetuximab and panitumumab). However, there are no cut-off levels for mutant RAS established in guidelines that could influence treatment efficacy, nor are there guidelines on which KRAS alleles confer resistance to anti-EGFR therapy. Additionally, studies indicate discordance in KRAS mutation status between primary and distant organ metastases, especially in pulmonary metastases [131,132].

A future approach may involve the repeated, real-time monitoring of RAS mutations via liquid biopsy, not only to guide treatment but also to detect emerging resistance mutations early, prior to disease progression [131].

##### NRAS Mutation

Current evidence suggests that patients with KRAS and NRAS mutations share similar clinical and pathological characteristics. However, those harboring NRAS mutations represent a distinct molecular and clinical subgroup of metastatic colorectal cancer (mCRC). Approximately 3–5% of CRC patients exhibit mutations in exons 2, 3, or 4 of the NRAS gene. These patients typically show a poor response to anti-EGFR therapy, and due to this lack of efficacy, the European Medicines Agency does not recommend the use of anti-EGFR agents in individuals with NRAS mutations [117,133,134].

##### BRAF Mutation as Predictive Marker

BRAF mutations (BRAFmt) are present in approximately 15% of primary colorectal cancers (CRCs) and in about 4–10% of patients with metastatic CRC (mCRC), and are associated with a poor prognosis [133].

The BRAF V600E mutation (BRAF V600Emt) represents up to 90% of all BRAF mutations and predicts less effect from anti-EGFR therapy. Observations from studies indicate that inhibiting mutant BRAF induces EGFR feedback activation. So, the recommendation for BRAF-mutant colorectal cancer is to combine anti-EGFR antibodies, BRAF inhibitors, and MEK inhibitors [120]. Moreover, in about 20% of cases, it can also be associated with deficient mismatch repair (dMMR) [129]. In contrast to BRAF V600Emt, atypical BRAF mutations (aBRAFmt) or non-V600Emt are rare but important to detect, as they are relevant to prognosis and treatment decisions, and co-exist with RAS mutation [129].

##### HER2 Gene Expression and HER2 Mutation

Amplification of the human epidermal growth factor receptor 2 gene (HER2 or ERBB2) is observed in approximately 5% of patients with metastatic colorectal cancer (mCRC), and it can be found in about 17% of those that also have KRAS mutations and in about 5% of those with RAS and BRAF wild-type tumors [126].

The National Comprehensive Cancer Network (NCCN) guidelines currently recommend HER2 amplification testing at the time of mCRC diagnosis, alongside assessment of KRAS/NRAS and BRAF status, to determine a patient’s eligibility for anti-HER2 therapy [135]. Overexpression of the HER2 gene in mCRC patients has been associated with worse prognosis and resistance to anti-EGFR therapy [126].

Regarding the prognostic and predictive roles of HER2 overexpression and mutation, inconsistent results have been reported across retrospective studies. A recent large analysis of randomized trials including patients with pMMR/MSS RAS/BRAF wild-type, untreated mCRC showed that those with HER2 amplification (HER2-positive) or HER2 mutation had a worse prognosis than those with negative HER2. Among HER2-positive patients, no difference in treatment effect was observed when comparing bevacizumab with anti-EGFR therapies in first-line chemotherapy for progression-free survival (PFS) [136,137].

#### 4.2.2. DNA Repair

##### MSI-H/dMMR in Localized and Metastatic CRC

Colorectal cancers characterized by high microsatellite instability (MSI-H) or mismatch repair deficiency (dMMR) constitute a molecular subtype with distinctive clinicopathological characteristics and a distinct prognosis that requires different treatment. In stage II colorectal cancer (CRC), about 20% of patients present with dMMR/MSI-H, whereas the proportion decreases to roughly 12% in stage III and is lowest in stage IV, at around 4% [138].

Studies have shown that the MSI-H status of a tumor is a favorable prognostic indicator in lower stages such as II-III TNM, with a potential benefit from adjuvant chemotherapy in stage II [139]. In the metastatic stage, MSI-H is a negative prognostic factor, potentially due to its association with BRAF mutations and its tendency to develop secondary-resistance-inducing mutations [140].

Current clinical guidelines recommend assessing mismatch repair (MMR) proteins by immunohistochemistry or molecular testing for microsatellite instability (MSI), both of which serve as key predictive biomarkers of immunotherapy responsiveness. This is especially evident with immune checkpoint inhibitors, as MSI-H/dMMR colorectal cancers (CRCs) are more immunogenic and respond better to immunotherapy than pMMR/MSS CRCs [131]. Studies like Keynote-177 and CheckMate-142 demonstrate the power of effective prediction [141,142]. For the dMMR/MSI-H patients eligible for curative surgical resection, neoadjuvant immunotherapy has shown high rates of response. In advanced dMMR/MSI-H or mCRC, pembrolizumab has demonstrated efficacy with lower toxicity than chemotherapy, and is therefore approved by the FDA as a first-line treatment [143,144].

Moreover, recent studies confirmed the feasibility of dual immunotherapy (PD-1 monoclonal antibody combined with cytotoxic T-lymphocyte-associated protein 4 (CTLA-4) monoclonal antibody therapy) with or without cetuximab [129]. Another dual adjuvant therapy for locally advanced dMMR CRC demonstrated promising results in the phase 2 NICHE-3 trial, which investigated the combination of PD-1 monoclonal antibodies with lymphocyte activation gene-3 (LAG-3) inhibitors [145].

Although MSI is recognized as a valuable predictive biomarker for immunotherapy in colon cancer, some MSI-H colon cancers exhibit intrinsic or acquired resistance to immunotherapy [125].

Regarding the need for rapid testing of MSI status, novel techniques that include advances in computational pathology, along with emerging applications of artificial intelligence (AI) in medicine, are being developed and reported in studies with promising accuracy in predicting MSI status [139].

##### TMB as a Predictive Biomarker

Tumor mutational burden status (TMB) is another promising and potentially valuable biomarker, capable of predicting responses to immune-checkpoint-based immunotherapy. Since it quantifies the number of mutations in the neoplastic cells’ genome and the neoantigen load that can activate the immune response, higher TMB correlates with a good response to immune checkpoint inhibitors in many studies across various cancer types [146]. The KEYNOTE-158 phase II trial showed that a variety of CRC patients with high TMB may demonstrate a strong tumor response to pembrolizumab monotherapy [147]. Therefore, the FDA approved the anti-PD-1 antibody pembrolizumab for CRC patients with high TMB (more than 10 mut/Mb) who had not responded to a prior therapy, advancing a personalized approach to medicine.

TMB is associated with other established biomarkers, such as microsatellite instability status, because it represents one of the several mechanisms that can lead to an elevated tumor mutational load. Prior studies have shown that TMB predicts response to immune-checkpoint-based immunotherapy in patients with MSI-H. For example, stratifying patients by TMB load may help identify new subgroups [148]. Furthermore, in microsatellite-stable (MSS) CRC with more than 10 mut/Mb, the response to immune checkpoint inhibitors is limited, indicating a prevalent immunosuppressive tumor microenvironment and the scarcity of neoantigens in MSS tumors [149]. Even if it seems promising, for introducing the TMB into clinical practice, there is a need for a calibrated method and established, satisfactory cut-offs for TMB levels to integrate with other markers for immunotherapy indications [85].

Moreover, studies have examined methods that combine multiplex PCR with machine learning algorithms to forecast immunogenicity and neoantigen load, providing estimates of TMB and immunological response [150,151]. Future strategy may include measuring TMB over time with liquid biopsy, a less invasive approach, and combining it with multimodal omics to stratify patients who can make the most of immunotherapy [150]. Current studies indicate that TMB alone is unlikely to identify eligible patients for immunotherapy, but it can be incorporated into diagnostic assessment and tumor staging [85].

##### POLE/POLD1 (Polymerase Epsilon/Delta) Mutations

The POLE and POLD1 genes encode enzymes involved in DNA replication and repair, but their tumor-associated mutations in CRC remain poorly understood. So far, studies have revealed that mutations in POLE/POLD1 impair DNA replication, producing a high tumor mutational burden that occurs independent of deficient mismatch repair (dMMR) and microsatellite instability status (MSI-H). The importance of these mutations in CRCs has recently become a real interest of clinical research. Based on limited data, POLE gene mutations appear to be an accurate molecular marker for predicting survival and dissemination in some solid cancers, including CRC [152]. Tumors with POLE/POLD1 mutations can have over 100 mutations per megabase and are associated with MSS. Most current studies have linked this ultra-mutated phenotype to increased sensitivity to immune checkpoint inhibitors (ICIs), likely due to heightened neoantigen generation [152,153]. Surprisingly, mutations in POLD1 gene in MSS tumors redefine the efficacy of ICIs independent of general microsatellite instability pathways [152,154].

Patients with metastatic CRC (mCRC) carrying mutations in POLE/POLD1 represent a rare (<1%) subtype with more than 100 mutations/Mb, so data on the administration and efficacy of immune checkpoint inhibitors (ICIs) are scarce compared with those with MSI-H [145]. Some retrospective studies showed better outcomes than patients with MSI-H treated with ICIs, even among those with extensively pretreated disease [155]. Several clinical trials are studying the potential of POLE/POLD1 mutation in MSS mCRC with high TMB, especially their role in predicting ICI response (NCT05103969) [154].

Along with their role in cancer progression, POLE/POLD1 mutations also serve as promising predictive biomarkers for immunotherapy response, expanding and reshaping the therapeutic landscape in precision oncology. One limiting factor in clinical practice is the low incidence of mutations, highlighting the need for prospective trials to validate their effectiveness as independent markers [156]. Integrating with other established biomarkers into predictive panels for immunotherapy can improve the effective selection of patients.

#### 4.2.3. Epigenetic Biomarkers—DNA Methylation, Histone Modifications, and Non-Coding RNA Changes

Among somatic mutations and altered genomic profiling, there is an increasing interest in epigenetic factors as predictive biomarkers in CRC in recent years, even though research in this area is still relatively limited compared with the more extensive studies examining their roles in early detection and prognosis [157].

##### DNA Methylation

Many studies have demonstrated that DNA methylation changes promise to be valuable biomarkers for diagnosis, as well as for prognostic information and treatment efficacy. Among the many DNA methylation-based panel markers studied for diagnosing CRC with high sensitivity, some can predict therapy response [158].

Beyond the diagnostic value of methylated SEPT9 (mSEPT9), a study by Jiang H. et al. showed that decreases in mSEPT9 levels after chemotherapy (XELOX, FOLFOX, FOLFIRI) correlated with better treatment response and outperformed CEA in predicting outcomes [159].

Given that LINE-1 (Long Interspersed Nuclear Element-1) activation through hypomethylation can disrupt genomic stability and contribute to cancer development, several studies investigated LINE-1 methylation levels and clinical outcomes in CRC patients. Hypomethylation of LINE-1 has been associated with unfavorable outcomes in stage II/III CRC patients [160]. Therefore, LINE-1 hypomethylation may be a useful biomarker for predicting disease recurrence and survival, especially in patients with stage II and III CRC. Moreover, a survival benefit for patients with stage II and III CRC with LINE-1 hypomethylation has been reported in a study investigating the potential benefit of adjuvant oral fluoropyrimidine therapy. Integrating LINE-1 methylation assessment into clinical practice could support more precise risk stratification and personalized treatment planning.

ERCC1 (Excision Repair 1, Endonuclease Non-Catalytic Subunit) and MGMT (O6-methylguanine-DNA-methyltransferase) are two repair enzymes related to oxaliplatin- and alkylating-based treatment that could predict FOLFOX resistance. Jamai D. et al. reported that methylation of ERCC1 and MGMT was associated with a less favorable prognosis and greater sensitivity to FOLFOX chemotherapy [161].

Recently, promoter methylation of the checkpoint with forkhead and ring finger domains (CHFR) gene has been associated with a good response to irinotecan-based therapy in CRC patients with advanced or metastatic disease. Hagiwara et al. demonstrated that CHFR promoter methylation predicted a stronger response to irinotecan-based therapy and longer progression-free survival (PFS), highlighting its potential as a treatment-response biomarker [162].

##### miRNAs

The prognostic and predictive value of aberrant expression of individual miRNAs and miRNA panels in CRC has been widely investigated. Horak et al. showed that low tumor miR-140 was linked to metastatic CRC and shorter survival in patients treated with surgery, while restoring miR-140 reduced cell proliferation and increased oxaliplatin sensitivity [163]. Regarding recurrence rates, elevated miR-21 in plasma or exosomes was strongly associated with CRC recurrence, with markedly higher recurrence rates and worse overall survival (OS) [156]. Similarly, high miR-4442 levels predicted early recurrence [158,164].

Moreover, several studies have highlighted the utility of miRNAs for assessing chemoresistance. In a study of CRC patients with advanced stage (III–IV), a plasma exosomal miRNA panel (miR-100, miR-92a, miR-16, miR-30e, miR-144-5p, let-7) effectively distinguished patients with oxaliplatin resistance, outperforming CEA and CA19-9 [158]. Another miRNA panel (miR-106a, miR-484, and miR-130b) analyzed by Kjersem et al. in 24 metastatic CRC patients found that these miRNAs were significantly elevated in non-responders to oxaliplatin-based therapy [165]. Similarly, Gherman et al. showed that higher exosomal levels of miR-92a-3p and miR-221-3p may signal resistance to first-line chemotherapy and correlate with reduced survival [166]. However, most evidence comes from preclinical studies, highlighting the need for larger clinical trials to confirm their relevance [167].

A distinct set of up- or downregulated miRNAs has been shown to predict response to neoadjuvant therapy: miR-145 (targeting TRIAP1, EGFR, and BAK1), miR-223 and miR-1246 (both targeting KRAS), and miR-622 (targeting TP53, KRAS, HIF1A, VEGFA, EGFR, and MKI67) [127]. Also, miR-548I1 was identified as upregulated in a cohort of stage III CRC patients with poor prognosis undergoing adjuvant chemotherapy [168].

In a CRC cell line study, Zhang W. et al. found that miR-31 decreased in a dose-dependent manner after radiation, regulating CRC radiosensitivity by inhibiting STK40, suggesting both as potential radiotherapy-response markers [169].

##### Long Non-Coding RNAs (lncRNAs)

Expression of numerous unbalanced lncRNAs in tumor tissue and blood has been associated with poor prognosis, but also with survival, metastasis, and tumor stage, emphasizing their potential as prognostic and predictive biomarkers [167]. Shen X et al. reported that serum lncRNA differentiation antagonizing non-protein coding RNA (DANCR) expression rises with advancing TNM stage and decreases after surgery or chemotherapy, but increases again at recurrence, suggesting its usefulness as a predictive and monitoring biomarker [170].

Fan C et al. showed that lncRNA MALAT1 (Metastasis-Associated Lung Adenocarcinoma Transcript 1) is overexpressed in oxaliplatin-resistant tumors, and its knockdown restored oxaliplatin sensitivity via the miR-324-3p/ADAM17 axis [171]. Additionally, lncRNA LUCAT1 (Lung-Cancer-Associated Transcript 1) is upregulated in CRC tissues and promotes tumor cell proliferation, as well as resistance to 5-fluorouracil (FU) and oxaliplatin [172]. In a recent study, lncRNA SNHG7 (Small Nucleolar RNA Host Gene 7) was similarly implicated in anlotinib resistance, a multikinase angiogenesis inhibitor, with higher expression in resistant CRC cell lines and reduced viability and colony formation after SNHG7 silencing [173].

In a cohort with advanced CRC patients receiving neoadjuvant therapy before surgery, Zhu Z et al. identified high lncRNA GAS6-AS1 (GAS6 Antinsense RNA 1) expression in CRC tissue, correlated with poor survival. Also, in vitro and in vivo studies identified GAS6-AS1 as a key driver of 5-fluorouracil resistance, with high GAS6-AS1 expression being correlated with poorer treatment response and reduced disease-free survival, while knockdown reversed these effects [174].

#### 4.2.4. Liquid Biopsy and Its Extensive Clinical Utility

In the last few decades, liquid biopsy has emerged as a transformative tool for CRC management. As a non-invasive technique, liquid biopsy has a great potential to characterize the tumor biology through biomarkers like cell-free nucleic acids (cfNAs), circulating tumor DNA (ctDNA), extracellular vesicle DNA (evDNA), circulating tumor cells (CTCs), microRNAs (miRNAs), long non-coding RNAs (lncRNAs), and circular RNAs (circRNAs). This valuable technique has multiple applications in detecting CRC, monitoring, and guiding the treatment selection. Therefore, ctDNA-based tests are available and recommended by international oncology societies for implementation in clinical practice [10].

Circulating cell-free nucleic acids (cfNAs) found in the blood plasma and serum of CRC patients have been proven to detect the disease. Several studies have demonstrated that the fragmentation patterns of cfDNA can mirror underlying epigenetic regulation, making it a marker for cancer screening. With rapid technological progress, cfDNA detected in blood samples is now regarded as a reliable indicator of disease progression, from initial tumor development to recurrence [175].

Lately, studies have shown that the cfDNA and cfDNA integrity index—defined as the ratio of long to short DNA fragments—are valuable biomarkers for detection and prognosis monitoring in patients with CRC [176]. Therefore, a small retrospective cohort study evaluating the need for neoadjuvant chemotherapy in locally advanced CRC found that, using machine learning, the cfDNA integrity index could be a promising biomarker for therapy efficacy. Given the lack of dependence on genetic mutations and the cost-efficiency, the integrity index offers a real-time, non-invasive method to monitor the response to chemotherapy, enabling prompt detection of non-responders or recurrence. In these situations, adjusting the chemotherapy regimen, adding radiotherapy, or advancing the timing of surgery could help reduce unnecessary exposure to treatments that are unlikely to be effective [118].

Moreover, in metastatic CRC with RAS mutations, a small study showed that plasma cfDNA analysis can predict outcome when bevacizumab is added to standard chemotherapy. Therefore, findings indicate that assessing chromosomal instability (CIN) in cfDNA can be a valuable tool for predicting outcomes and response to bevacizumab combination therapy, with CIN-high tumors showing better survival compared to those with low levels. Numerous studies have demonstrated that identifying genetic and non-genetic alterations in cfDNA—including copy number alterations, nucleosome patterns, and methylation signatures—has substantial value for cancer detection, prognosis, and treatment surveillance [177].

In addition to cfDNA, circulating tumor DNA (ctDNA) has emerged as a valuable biomarker for CRC management. Beyond its prognostic ability to detect post-surgical molecular residual disease and forecast recurrence, circulating tumor DNA (ctDNA) can guide treatment selection [178]. So, in the early stage (I-III), it may serve as a temporal indicator of adjuvant therapy. Early findings from the DYNAMIC study in stage II colon cancer demonstrated that patients with positive ctDNA had the greatest benefit from adjuvant treatment compared with those with the standard approach. Recurrence-free survival was comparable between patients managed with ctDNA-guided therapy and those treated with standard care. Additionally, persistent ctDNA after adjuvant chemotherapy was associated with a high likelihood of disease recurrence [108].

In metastatic CRC, ctDNA is applicable for diagnosis, with studies demonstrating strong concordance between the tumor molecular signature detected by ctDNA and that by tissue biopsy. Moreover, monitoring ctDNA during treatment could inform early response and prognosis and detect therapy resistance during disease progression [179]. Therefore, studies support the value of dynamically tracking ctDNA levels, with high accuracy in detecting disease progression through elevated levels. Moreover, it can detect the possibility of conversion of an initially RAS-mutant tumor in tissue to RAS wild-type tumor [180].

Additionally, a validated liquid biopsy assay that sequences hundreds of cancer-related genes, FoundationOne Liquid CDx, has recently been approved by the FDA as a companion diagnostic for guiding the use of encorafenib plus cetuximab in mCRC patients with a BRAF V600E mutation, as well as entrectinib for rare NTRK1–3 fusions in solid tumors [10].

Contrary to traditional biomarkers and tissue biopsies, ctDNA is non-invasive, providing real-time and meaningful information about tumor load and molecular heterogeneity [178]. Therefore, liquid biopsy could replace the tissue-based TMB assessment, offering particular value for tumors that are difficult to access or exhibit significant spatial heterogeneity in their mutational landscape. Blood TMB (bTMB) quantifies somatic mutations in ctDNA and reflects the overall mutational burden from both primary and metastatic sites. In recent studies, assays such as GuardantOMNI and PredicineATLAS have validated the bTMB measurement, demonstrating good concordance with tissue-based TMB across multiple cancer types [150]. As the field continues to advance, further research will be essential to clarify and optimize the use of ctDNA in guiding treatment decisions across additional clinical contexts.

Nonetheless, significant challenges remain, such as standardizing testing methods, enhancing sensitivity for early-stage disease, and promoting broader clinical implementation [178]. The main molecular biomarkers currently used in clinical practice, along with their prognostic and predictive significance, are outlined in Table 3.

## 5. Emerging Directions and Key Challenges

### Novel Liquid Biopsy-Linked Biomarkers for CRC

Recently, extracellular vesicle DNA (evDNA) as a new target for liquid biopsy has gained attention in diagnosis and prognosis in CRC. Unlike ctDNA, it is continuously shed from cancer cells, incorporated into extracellular vesicles along with RNA and proteins. Data from a few studies with mCRC patients showed that evDNA has better sensitivity in identifying KRAS mutations than tissue biopsy and ctDNA at diagnosis and during treatment monitoring [184,185]. In a recent study on non-metastatic and metastatic CRC, the superiority of evDNA over ctDNA was demonstrated in detecting KRAS mutations, especially in stage II and III. This highlights its important clinical value in testing not only from the tissue, but also from the blood, establishing the use of anti-EGFR antibodies [194].

EVs represent a transformative avenue in CRC research, offering non-invasive diagnostic and prognostic opportunities and novel therapeutic avenues. By combining advanced technologies with a deeper understanding of EV regulatory mechanisms, they can drive progress in precision medicine, enhancing patient outcomes [195].

Circular RNAs (CircRNAs) are another set of key regulators of gene expression, expressed in tissues, blood/serum, and exosomes of CRC patients, and recognized as drivers of tumor initiation and progression. Emerging studies link some dysregulated circ RNAs with the progression of CRC, making them potential non-invasive diagnostic markers and a therapy target. So far, studies have described more than 70 circRNAs with elevated levels implicated in tumor formation and progression [196].

Several circRNAs, including circ1662, circPACRGL, and circ-KLDHC10, are elevated in patients with CRC. Due to their high stability, circRNAs are promising for qRT-PCR-based diagnosis in liquid biopsy. Others, such as circALG1, correlate with metastatic disease, further supporting their prognostic value [186]. Similarly, circ3823 promotes proliferation, metastasis, and angiogenesis, predicting a poor prognosis for CRC patients [187]. Moreover, circRNA profiling has identified circRNA_0001178 and circRNA_0000826 with high diagnostic accuracy for liver metastases from CRC [188]. Additionally, elevated circPTK2 levels in CRC tissues and serum were positively associated with metastasis, advanced clinical stage, and chemoresistance [189]. Likewise, increased circ5615 expression in CRC tissues showed a strong correlation with higher disease stage and poorer patient prognosis [190]. Collectively, these findings position circRNAs as valuable diagnostic and prognostic biomarkers for CRC.

Several circRNAs—such as circRS-122, circ_001680, circ_0002813, circ_101277, circ_0000236, and circ-ZEB1—have been linked to resistance to chemotherapies including 5-FU, oxaliplatin, cisplatin, and irinotecan [191,196]. Others, like circIFNGR2 and circLHFPL2, are associated with resistance to targeted agents such as cetuximab and MEK inhibitors [192,193]. These findings suggest that circRNAs may serve as valuable predictors of therapeutic resistance in CRC.

Although advances in RNA sequencing have improved circRNA detection, challenges persist—including reliably distinguishing circular from linear transcripts and clarifying their biological functions. Many disturbances of circRNAs have been linked to chemoradiation resistance, and overall, circRNAs show strong promise for CRC early detection, prognosis, and therapy. However, clinical translation remains limited by the need for standardized assays, large-scale validation, and deeper mechanistic insight. Integrating multi-omics data with AI-driven analytical models may accelerate the development of circRNA-based diagnostics and support more personalized, effective CRC treatment [197].

Exosomes, vesicles released by tumor cells, fibroblasts, immune cells, and endothelial cells within the TME, are increasingly studied as biomarkers in liquid biopsy and as delivery vehicles for therapeutic agents, due to their systemic access and molecular stability. Nevertheless, clinical translation is hindered by challenges related to cell-specific targeting and unintended effects on non-tumor tissues. Longitudinal studies that monitor the evolution of the tumor microenvironment (TME) during disease progression and therapy will be essential for predicting resistance mechanisms and developing adaptive, sustainable treatment strategies [8]. Prognostic classifiers based on TME patterns have also been developed to predict disease progression using mRNA, microRNA, long non-coding RNA, and other TME-associated genes. A recent study evaluated the prognostic potential of a tumor microenvironment (TME) molecular signature for colorectal cancer (CRC) using machine learning regression methods. The TME-based prognostic signature (TPS), comprising an 11-gene molecular profile, demonstrated superior prognostic performance, indicating significant potential for future clinical applications [198].

Therefore, liquid biopsy offers a non-invasive approach for monitoring biomarkers such as RAS. Expanding access to extensive molecular profiling through cost-effective alternatives, such as digital droplet PCR analysis of hypermethylated genes, enables future clinical applications, even in mCRC [75]. Thus, by implementing validated, high-sensitivity ctDNA assays, the care for patients with metastatic colorectal cancer could be enhanced by providing earlier treatment and a shorter time to therapy initiation [199].

Neoantigens, tumor-specific antigens caused by somatic mutations, have emerged as key targets for personalized immunotherapy. Because they are recognizable by the immune system, they form the basis for individualized vaccines and adoptive T-cell therapies. Neoantigen characterization depends on next-generation sequencing (NGS) to detect non-synonymous tumor mutations, followed by computational tools that predict which mutated peptides can bind MHC molecules and elicit an immune response. This enables precise identification of actionable neoantigens, supporting the development of highly personalized cancer immunotherapies [150].

## 6. Discussion

Colorectal cancer (CRC) remains one of the leading causes of cancer-related morbidity and mortality worldwide, despite significant advances in screening, surgical techniques, and adjuvant therapies [5]. Prognosis and treatment decisions in CRC have traditionally relied on clinicopathological parameters such as tumor–node–metastasis (TNM) stage, histological grade, and lymphovascular invasion. Although these factors provide a valuable framework for risk stratification, they often fail to fully capture the biological heterogeneity of the disease [200]. Patients within the same clinical stage can experience markedly different outcomes, underscoring the limitations of morphology-based classifications.

This gap has stimulated the search for molecular tools capable of refining prognostic assessment and predicting therapeutic response. The identification of specific genetic and epigenetic alterations has redefined the understanding of CRC progression as a multistep molecular process involving the accumulation of mutations in oncogenes, tumor suppressor genes, and DNA repair pathways [201]. Classic molecular features such as microsatellite instability (MSI), CpG island methylator phenotype (CIMP), and mutations in KRAS, BRAF, TP53, and APC have provided important insights into tumor biology and patient outcomes [202]. However, their prognostic accuracy remains limited when used in isolation.

In recent years, advances in tumor knowledge have improved screening methods. New non-invasive or minimally invasive techniques have improved the early detection of CRC or even precancerous lesions. New stool-based tests with good sensitivity that detect DNA mutations, DNA methylation, metabolic enzymes, or blood have increased adherence to screening programs compared with traditional colonoscopy. With higher costs, but non-invasive, blood-based tests identify genetic, epigenetic, or proteomic tumor changes, circulating tumor cells (CTCs), and cell-free DNA (cfDNA), showing good potential for CRC detection but not for advanced adenomas [6]. To improve the sensitivity of the first approved test for the methylated SEPT9 gene, the latest tests combine complementary molecular markers and techniques, with promising results. Given its many advantages, liquid biopsy is a promising method for introduction into screening programs [60].

To assess tumor aggressiveness and patient survival, numerous prognostic markers have been identified, including genomic profiling that identified key mutations. Even though they correlate with disease invasiveness, they can provide information about potential therapeutic response and serve as predictive markers. Mutations in the RAS family (KRAS and NRAS), BRAF, and HER2 are recommended by clinical guidelines for metastatic CRC to help predict patient prognosis and guide treatment selection, given the resistance to anti-EGFR therapy among tumors harboring these mutations [76,78]. Importantly, no cut-off levels for treatment have been established, nor are guidelines available for which KRAS alleles confer resistance to therapy. It has also been reported that there are differences in KRAS mutation status between the primary tumor and metastases [113,114].

DNA repair deficiency markers, such as dMMR, MSI, and TMB, have implications for prognosis and response to certain therapies. Testing for MSI status is now mandatory in all CRCs, offering not only therapeutic guidance for MSI-H tumors but also prognostic value [77,181]. In the lower stage (II-III), MSI-H status is a positive prognostic factor and indicates the need for adjuvant therapy, while in the metastatic stage, it is a negative prognostic factor [127,128]. Importantly, treatment response in MSI-H CRCs can be affected by reported immunotherapy resistance [125]. By quantifying overall genomic alterations, TMB has proven to be a biomarker associated with a good outcome in those with MSI-H or POLE/POLD1 mutations and to indicate the use of immune checkpoint inhibitors [85].

In addition, epigenetic and transcriptomic alterations, such as aberrant DNA methylation and non-coding RNAs (ncRNAs)—including microRNAs (miRNAs) and long non-coding RNAs (lncRNAs)—have been correlated with tumor progression and prognosis, as well as with some treatment resistances [86,87,88,89,90]. Many DNA methylation changes have not only diagnostic power, but also prognostic and predictive values. Methylated SEPT9 (mSEPT9) is a diagnostic biomarker (Epi proColon) and has been associated with better treatment response. Non-coding RNAs have been studied intensively in recent years, with numerous miRNAs demonstrating both prognostic and predictive power, but their clinical relevance has not yet been confirmed in large studies.

The revolutionary non-invasive method, liquid biopsy, has not only screening applications but can also provide valuable prognostic information by analyzing materials detached from the tumor in blood. Components such as cell-free DNA (cfDNA), circulating tumor DNA (ctDNA), circulating tumor cells (CTCs), and extracellular vesicles, such as exosomes, can support detecting of early recurrence, predict survival outcomes, and assess treatment response. Therefore, ctDNA has prognostic and predictive value, offering non-invasive, real-time molecular monitoring of disease. With new technologies that offer increased sensitivity and specificity, such as digital droplet PCR and next-generation sequencing (NGS), it can detect early disease recurrence and enable assessment of therapy response [112,113]. Also, cfDNA levels have been shown to be important in CRC care. They are associated with tumor aggressiveness, metastasis, and poor patient survival [116,117].

With new biomarkers such as circular RNAs (circRNAs) and extracellular vesicle DNA (evDNA), liquid biopsy can be integrated into clinical practice for personalized treatment. EvDNA demonstrates greater sensitivity for KRAS identification than tissue biopsy and throughout therapy [167,168]. Dysregulated circRNAs have been shown to act as tumor initiators, making them potential diagnostic tools. Several circRNAs are implicated in tumor progression, metastasis, and chemoresistance, and show potential as prognostic and predictive biomarkers [169,170,171,172,173,174,175].

Consequently, there is an increasing demand for integrative molecular platforms that combine multiple biomarkers and high-throughput “omics” data to generate individualized prognostic models. Such tools have the potential to complement or even surpass conventional staging systems by providing more precise estimates of recurrence risk, overall survival, and response to targeted or immunotherapeutic agents [203]. As personalized oncology continues to evolve, the development and validation of robust molecular prognostic tools have become a central objective in the management of CRC.

Despite substantial advances in the discovery and validation of molecular prognostic tools, their integration into clinical practice for CRC remains limited. Most biomarkers identified to date—whether genomic, epigenetic, transcriptomic, or immune-related—have demonstrated promising associations with survival outcomes, yet only a few have achieved consistent reproducibility, regulatory approval, and cost-effective clinical applicability. Translating these molecular insights into standardized prognostic models requires overcoming multiple scientific, technical, and logistical challenges.

A major limitation lies in the heterogeneity of study design and analytical methods across research cohorts. Differences in patient populations, tumor sampling, assay platforms, and data normalization often yield inconsistent results, hindering cross-study comparability. Moreover, tumor heterogeneity itself—both intertumoral and intratumoral—poses a significant obstacle to accurate prognostication. Molecular profiles can vary between primary tumors and metastases or evolve under therapeutic pressure, complicating the use of static biomarkers for long-term risk assessment.

Another barrier is the lack of standardization in molecular testing, including pre-analytical handling, assay calibration, and bioinformatic interpretation. For example, discrepancies in defining CpG island methylator phenotype (CIMP) or thresholds for tumor mutational burden (TMB) contribute to divergent prognostic conclusions. In addition, the high cost and technical complexity of next-generation sequencing and multi-omic analyses limit their widespread implementation, particularly in low- and middle-income healthcare systems.

From a clinical perspective, incorporating molecular prognostic tools into decision-making algorithms requires robust validation in large, prospective, and multicenter trials. While assays such as *Oncotype DX Colon* and *Immunoscore^®^* have achieved partial clinical adoption, the majority of candidate biomarkers remain confined to research settings. Furthermore, integrating molecular data with established clinicopathological factors and imaging modalities will be critical to building comprehensive and actionable prognostic frameworks.

As new therapies emerge, there is a need for accurate predictive biomarkers to help tailor treatment. Considering the numerous side effects of chemotherapy, identifying specific molecular tools allows recognizing, at the same time, potential non-responder patients [127]. Non-invasive, accurate, and real-time predictive biomarkers are needed in clinical practice to guide treatment selection as the disease evolves. Therefore, liquid biopsy and new technologies could help fill the gap in personalized CRC treatment. In this regard, a recently validated liquid biopsy, *FoundationOne Liquid CDx*, is indicated for the combination of encorafenib plus cetuximab in mCRC with BRAF V600E mutation [10].

## 7. Conclusions

The evolution of molecular prognostic tools in CRC reflects the broader transformation of oncology toward precision medicine. Traditional clinicopathological parameters, while essential for initial staging and treatment planning, fail to fully capture the molecular complexity and biological heterogeneity that drive disease behavior. Over the past two decades, advances in genomics, epigenomics, transcriptomics, proteomics, and immunology have enabled the identification of a wide range of biomarkers—ranging from classical mutations such as KRAS, BRAF, and TP53 to more sophisticated indicators such as microsatellite instability (MSI), DNA methylation patterns, non-coding RNAs, and circulating tumor DNA (ctDNA).

Molecular screening tools such as the multitarget stool DNA test, which relies on methylated DNA detection, show greater potential than FIT. However, their high cost continues to limit broad implementation.

Advances in genomic technologies have revealed a wide range of genetic and epigenetic alterations, establishing them as promising clinical markers for early diagnosis, screening, prognosis, and treatment response prediction in CRC. Combined biomarker panels, in particular, offer enhanced sensitivity and specificity, including in early-stage detection. Among methylation-based markers, SEPT9, NDRG4, BMP3, SDC2, SHOX2, and VIM, as well as LINE-1, ERCC1, MGMT, and CHFR, show strong diagnostic, predictive, or prognostic relevance. Several miRNAs and lncRNAs have also demonstrated significant clinical potential. Despite encouraging findings and strong diagnostic potential, many studies still require large, multicenter clinical validation, as small sample sizes limit their reliability. Even so, gene methylation analysis holds considerable promise as a future alternative to invasive or low-accuracy CRC screening methods, with the potential to significantly improve early detection and survival outcomes.

For a non-invasive approach, liquid biopsy has the potential to revolutionize CRC management through cfDNA, ctDNA, evDNA, and circRNA, but its integration into clinical practice must proceed cautiously until results from ongoing randomized trials and clear guidelines are available. Although significant advancements in liquid biopsy technology are promising, the costs for implementing it in clinical practice could be prohibitive. In metastatic CRC, ctDNA can support molecular selection for targeted therapy, and in resectable disease, ctDNA-guided MRD assessment is highly promising. Although the FDA has approved a liquid biopsy test for cancer screening, with some limitations, it should not replace established diagnostic tools. To maximize the clinical utility of ctDNA, there is a need for additional clinical trials and evidence-based protocols. Some successful models of biomarker integration, like Oncotype DX Colon and Immunoscore, have already proven to be prognostic tools for stage II and III CRC. FoundationOne Liquid CDX helps guide treatment even in advanced stages, and GuardantOMNI and PredicineATLAS have been validated as valuable genomic profiling tools for measuring blood TMB to support personalized therapy.

The future of personalized therapy will involve expanding biomarker panels to capture additional resistance pathways and integrating multi-omic approaches, such as combining ctDNA with transcriptomic, proteomic, and even metabolomic data. These methods provide a more complex molecular comprehension of tumor biology that could improve diagnostic and predictive evaluation.

However, despite their promise, routine clinical implementation remains limited by issues of standardization, cost, validation, and accessibility. To achieve widespread impact, future efforts must focus on harmonizing methodologies, expanding prospective validation, and developing cost-efficient assays suitable for clinical workflows. The integration of artificial intelligence and liquid-biopsy-based monitoring further holds potential to transform prognostic evaluation into a dynamic, real-time process.

**Figure 2:**
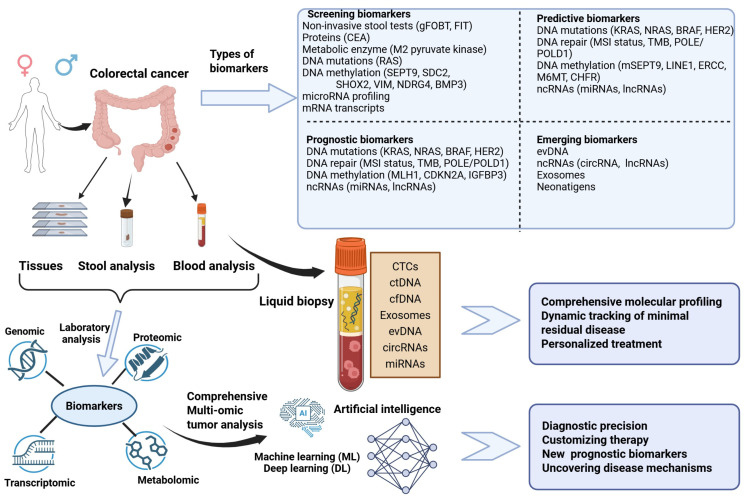
Schematic representation of colorectal cancer detection at any stage using stool analysis, tissue analysis, and liquid biopsy. Evaluation of molecules such as proteins, DNA, RNA fragments, circulating tumor DNA (ctDNA), and circulating tumor cells enables identification of genetic, epigenetic, transcriptomic, or proteomic tumor changes that serve as colorectal cancer biomarkers. Integration of multi-omic biomarkers with artificial intelligence and machine learning algorithms generates combined molecular panels that enhance early diagnosis, screening, prognosis, and prediction of treatment response in colorectal cancer. Created in BioRender. Ceafalan, L. (2026) https://BioRender.com/vko6ab7.

By overcoming current limitations, AI could greatly improve the precision and efficiency of cancer diagnostics, enabling more personalized and effective treatments. Continued progress in AI-driven computational pathology may further enhance the ability to predict the responses to immunotherapy from histology and support the discovery of new biomarkers and therapeutic options for CRC patients (Figure 2).

## 8. Future Directions

Screening methods that can detect CRC earlier, with good patient compliance, such as blood-based tests, can help reduce the disease’s high mortality rate. Although the blood-based tests that detect the small amount of ctDNA shed by tumor cells have low sensitivity, additional techniques have been investigated to improve detection. Therefore, combining specific DNA methylation markers, fragmentomics, proteomics, and new potential biomarkers such as evDNAs and circRNAs and integrating with AI analytical models could be an important step toward more customized patient care (Figure 2).

Prognostic models that combine epigenetic and transcriptomic profiles have a greater precision than single-gene or one-mutation-based assays. New technologies such as DNA methylation microarrays, RNA sequencing, and single-cell transcriptomics can provide additional information about the adaptive tumor cells’ microenvironment, with important implications for recurrence and therapy resistance. Moreover, liquid biopsy technologies, especially ctDNA, offer a comprehensive molecular profiling with a great capacity to provide dynamic tracking of minimal residual disease [103,104].

Even though the ctDNA-guided therapy has great potential for patient management and therapy adjustment, standardized cut-off levels and protocols are being developed from ongoing trials (UMIN000039205 (https://center6.umin.ac.jp/cgi-open-bin/icdr_e/ctr_view.cgi?recptno=R000044197, accessed on 23 February 2026); NCT05174169) for introduction into clinical prognostic models for CRC. Moreover, predictive models have incorporated multi-omic data, digital pathology, and radiomic features using artificial intelligence and machine learning. Also, the implementation of these models in clinical practice requires accurate validation in studies, access to technologies, and strong collaboration among clinicians, pathologists, and bioinformaticians. These advances could overcome treatment resistance and provide answers to many questions about tumor behavior. In this direction, the next strategy is real-time surveillance of RAS mutations via liquid biopsy to detect the emergence of resistance mutations before progression [131]. A promising biomarker, POLE/POLD1 mutations, could expand the field of personalized therapy by assessing response to immune checkpoint inhibitors independent of well-known microsatellite instability. Despite being rare mutations, they can improve the selection of patients by combining with established biomarkers in predictive panels for immunotherapy, such as dMMR status and TMB [139,141].

### 8.1. Emerging Biomarkers

Conventional biomarkers for CRC management have their limitations, so the increasing need for earlier diagnosis and better outcomes has led to the development of more efficient, non-invasive biomarkers. Studies on blood-based DNA biomarkers indicate that cfDNA, ctDNA, and extracellular vesicle DNA (evDNA) hold significant promise for early CRC detection. RNA-based biomarkers, including miRNAs, lncRNAs, and circRNAs, have also demonstrated encouraging diagnostic potential. Among protein biomarkers, new candidates such as Irisin and ANXA2 were noted when compared with traditional tumor markers [204]. Some individual biomarkers and multi-marker panels have emerged with the potential to enhance, or even surpass, current CRC screening methods, addressing the critical need for earlier and more accurate detection. Moreover, several of them could improve the prognosis of CRC patients by guiding treatment selection [205].

### 8.2. Advanced Technologies

Emerging techniques like multiplex immunohistochemistry (mIHC) and single-cell RNA sequencing offer deeper insight into genetic diversity and the tumor microenvironment in CRC, allowing the identification of high-accuracy screening biomarkers and the assessment of response to immunotherapy [125].

Lately, multi-omics data analysis has attracted attention in research. It provides a comprehensive understanding of the molecular changes underlying disease occurrence, progression, and metastasis by integrating genomic, transcriptomic, metabolomic, and epigenomic layers [206].

Omics technologies facilitate the identification of biomarkers for non-invasive early CRC detection, subtype classification, and prediction of disease stage, prognosis, and overall survival [207].

New panels that combine proteomic, genomic, and transcriptomic assays are identifying candidate markers in plasma or tissues, including cytokines, exosomal proteins, and enzyme variants, by clarifying the cellular movements and spatial changes in the primary tumor microenvironment and the metastatic sites. Moreover, artificial intelligence (AI)-driven integration of multi-omic data (genomic, epigenomic, proteomic, metabolomic) is showing potential for improving diagnostic precision and customizing therapy [207].

Based on multi-omic integration with AI, tumor antigens and immune gene targets can be identified for combined and personalized immunotherapy and cancer vaccines, offering a novel strategy to improve patient outcomes [208]. Therefore, research studies report promising high precision in rapid prediction of MSI status by integrating new computational pathology techniques with AI [139].

In CRC, AI has multiple applications, such as enhancing polyp detection during colonoscopy, improving histopathologic classification, and predicting outcomes from clinical and molecular data [209].

Machine learning (ML) has shown high potential for identifying prognostic biomarkers and uncovering disease mechanisms from omics data [210]. For instance, Liu et al. integrated multiple ML models with immune-related cell analyses to identify prognostic lncRNAs in CRC [211]. Machine learning models like EML, ANN, DNN, and SVM exhibit high diagnostic accuracy for CRC detection. However, they need further rigorous external and prospective validation before they can impact clinical decision-making [209].

Moving forward, research should aim to broaden the range of actionable biomarkers, address resistance to targeted treatments, and enhance worldwide access to molecular diagnostic technologies.

## Figures and Tables

**Table 1:** Stool-based tests for colorectal cancer detection.

Test	Principle	Advantages	Limitations/Performance	Examples/Notes	References
Guaiac-Based Fecal Occult Blood Test (gFOBT)	Detects peroxidase activity of heme (blue color change)	Widely available, inexpensive, established in population screening	Requires multiple samples; influenced by diet & meds; sensitivity ~50–70% (CRC), 10–30% (adenomas)	Historic use in population screening	[20,21]
Fecal Immunochemical Test (FIT/iFOBT)	Antibodies detect human hemoglobin in stool	Higher sensitivity vs. gFOBT; no dietary restrictions; single sample	Detects only bleeding lesions → may miss non-bleeding adenomas/serrated lesions; sensitivity ~79%, specificity ~94%	First-line screening test in many guidelines	[20,22]
Fecal Tumor M2-Pyruvate Kinase (M2-PK)	Detects tumor-specific dimeric M2 isoform of pyruvate kinase in stool	Independent of bleeding; may complement FIT	Variable performance; not yet in guidelines; sensitivity ~80% (CRC), lower for adenomas	Potential adjunctive marker	[23]
Multitarget Stool DNA (mt-sDNA)	Detects KRAS mutations, methylated DNA (NDRG4, BMP3), β-actin, + fecal Hb	High sensitivity for CRC & advanced adenomas	Sensitivity 92.3% vs. 73.8% (FIT); specificity ~86.6% (lower than FIT); costly; complex lab processing	Cologuard^®^ (Exact Sciences Corporation, Madison, WI, USA) (FDA-approved, CMS-covered); Cologuard plus^®^ (FDA-approved, 2024)	[24,25]
Emerging Stool DNA Methylation Panels	Detects aberrant methylation in promoters of tumor suppressor/signaling genes	Potentially cost-effective; can use single or multiplex biomarkers	Sensitivities > 85% (CRC), lower for adenomas; still under validation	Genes: SEPT9, SDC2, VIM	[17,26]
Stool microRNA (miRNA) Profiling	Detection of tumor-derived miRNAs shed into stool (e.g., miR-92a, miR-21)	Non-invasive; stable in stool; reflects tumor biology	Variable performance; sensitivity 71–89%, specificity 65–81%; needs multicenter validation	miR-92a, miR-21 early studies; fecal miRNA signatures validated with NGS in 2023	[27,28,29]
Stool RNA Expression Panels	Multiplex detection of host mRNA markers in stool (mt-sRNA)	Broad marker coverage; standardized kit; phase 3 data	Sensitivity > 80% for CRC; ~50% for advanced adenomas; requires specialized assay	ColoSense (mt-sRNA) test; phase 3 JAMA 2023 trial; CRC-PREVENT clinical trial; FDA-approved in 2024	[30]

**Table 2:** Overview of principal blood-based biomarker assays for colorectal cancer screening.

Test/Marker	Principle	Advantages	Limitations/Performance	Notes	References
Epi proColon^®^ (SEPT9)	Plasma DNA methylation of SEPT9 promoter	Minimally invasive, FDA-approved	Sensitivity 68–72%, specificity 79–82%; poor adenoma detection	Approved for CRC screening in US and EU	[35]
ColoDefense	cfDNA methylation of SDC2 + SEPT9	Improved sensitivity vs. SEPT9 alone	Not yet FDA-approved	Promising results in Asian cohorts	[36]
CEA, CA19-9	Serum protein biomarkers	Widely available, low cost	Low sensitivity/specificity for early CRC	Used for monitoring, not screening	[37]
Guardant Shield™	cfDNA methylation + fragmentomics + mutations	High accuracy, pan-cancer potential	Expensive, early-stage validation	Ongoing clinical trials (ECLIPSE)	[38]
Multigene NGS panels (KRAS, APC, TP53)	Mutation detection in cfDNA	Personalized, high specificity	Not sensitive for early disease	Research and MRD monitoring use	[39]

**Table 3:** Overview of principles molecular biomarkers and their prognosis and predictive values.

Biomarker	Prognosis	Predictive	Observations	Detection in Liquid Biopsy	Reference
KRAS mutation	unfavorable	yes	Resistance to anti-EGFR therapy Lower response to third-line therapy (trifluridine/tipiracil (FTD/TPI))	yes	[74,130,181]
NRAS mutation	unfavorable	yes	Resistance to anti-EGFR therapy	yes	[133,134,135]
BRAF mutation	unfavorable	yes	BRAF V600Emt resistance to anti-EGFR therapy; may be associated with dMMR	yes	[131,182]
HER2 mutation	unfavorable	yes	Resistance to anti-EGFR therapy; indication for anti-HER2 therapy May be associated with KRAS mutation	yes	[126,135]
MSI/dMMR	favorable (localized CRC) unfavorable (advanced CRC)	yes	Sensitivity to ICIs (PD-1 inhibitors) Some MSI-H exhibit resistance to immunotherapy	yes	[82,83,141]
TMB	favorable (MSI-H/POLE/POLD1 mutation tumors)	yes	Sensitivity to ICIs; TMB-high at ~10 mutations per megabase	yes	[85,86,147]
POLE/POLD1 mutation	unfavorable	yes	Sensitivity to ICIs Associated with MSS Low frequency	yes	[152,153]
Methylated SEPT9	unfavorable	yes	Predict survival Monitoring of recurrence Promising treatment monitoring	yes	[148,183]
Hypomethylation of LINE-1	unfavorable	yes	Potential benefit for adjuvant therapy	yes	[160]
Methylation of ERCC1, MGMT	unfavorable	yes	Sensitivity to FOLFOX chemotherapy	yes	[161]
miRNAs miR-21, miR 92a, miR-200c upregulated, miR-143, miR-145	unfavorable	yes	Resistance to chemotherapy	yes	[95,96,97,98]
lnRNAs HOTAIR MALAT1 CCAT1 LUCAT1	unfavorable		Resistance to chemotherapy	yes	[99,100,101]
cfDNAs	unfavorable	yes	Treatment monitoring Recurrence monitoring	yes	[118]
ctDNAs	good	yes	MRD indicator Monitoring of recurrence Detects genomic alterations, tumor burden Early relapse detection Personalized therapy Treatment monitoring Detects drug resistance	yes	[106,107,109,111]
evDNAs	yes	yes	Better sensitivity to detect KRAS mutation; early CRC detection	yes	[184,185]
circRNAs	yes	yes	Resistance to chemotherapy	yes	[186,187,188,189,190,191,192,193]

ICIs—checkpoint inhibitors; MRD—minimal residual disease; BRAF-B600Emt—BRAF V600E mutation; FOLFOX—leucovorin (folinic acid), fluorouracil (5FU), and oxaliplatin.

## Data Availability

No new data were created or analyzed in this study. Data sharing is not applicable to this article.

## Abbreviations

The following abbreviations are used in this manuscript:

BRAF	B-Raf Proto-Oncogene, Serine/Threonine Kinase
KRAS	Kirsten Rat Sarcoma Virus
NRAS	Neuroblastoma RAS Viral Oncogene Homolog
HER2	Human Epidermal Growth Factor Receptor 2
APC	Adenomatous Polyposis Coli
PCR	Polymerase Chain Reaction
NDRG4	N-myc Downstream-Regulated Gene 4
BMP3	Bone Morphogenetic Protein 3
UICC	Union for International Cancer Control
AJCC	American Joint Committee on Cancer
ESMO	European Society For Medical Oncology
NCCN	National Comprehensive Cancer Network
EGFR	Epidermal Growth Factor Receptor
PIK3CA	Phosphatidylinositol-4,5-Bisphosphate 3-Kinase Catalytic Subunit Alpha
MEK	Mitogen-Activated Protein Kinase Enzymes
XELOX	Capecitabine and Oxaliplatin
FOLFOX	Folinic Acid, Fluorouracil and Oxaliplatin
FOLFIRI	Folinic Acid, Fluorouracil and Irinotecan
TRIAP1	TP53-Regulated Inhibitor of Apoptosis 1
BAK1	BCL2 Antagonist/Killer 1
HIF1A	Hypoxia-Inducible Factor 1 Subunit Alpha
VEGFA	Vascular Endothelial Growth Factor A
MKI67	Marker Of Proliferation Ki-67
STK40	Serine/Threonine Kinase 40
